# Beyond database training: reconceptualizing information literacy and reading competence as a shared cognitive architecture in medical education

**DOI:** 10.5195/jmla.2026.2385

**Published:** 2026-10-01

**Authors:** Mercedes Byrd, James K. Martin, Alexis Karas

**Affiliations:** 1 byrdm@rowan.edu, Instruction, Reference, & Research Support Librarian, Rowan-Virtua School of Osteopathic Medicine Health Sciences Library, Stratford, NJ; 2 jamesm1@gwu.edu, STEM Educational Developer, The George Washington University, Washington, DC; 3 karasa16@rowan.edu, Library Specialist II, Rowan-Virtua School of Osteopathic Medicine Health Sciences Library, Stratford, NJ

**Keywords:** Information literacy, medical education, metacognition, cognitive load theory, epistemic development, health sciences librarianship

## Abstract

Medical librarians have long observed that medical students who struggle with reading complex textbooks also demonstrate weak information literacy skills. For decades, these have been treated as separate problems requiring separate solutions—offering reading workshops and database training sessions with limited lasting impact. This commentary proposes a reconceptualization: textbook literacy and information literacy are not sequential competencies but rather parallel manifestations of the same underlying cognitive architecture. Drawing on research in medical education, cognitive psychology, and educational neuroscience, this commentary argues that both literacies depend on three interconnected cognitive capacities: metacognitive self-regulation, working memory management, and epistemic sophistication. Understanding this shared foundation has profound implications for how health sciences librarians design interventions, plan instruction, and position themselves as essential contributors to medical education. This commentary presents a framework and outlines practical implications for library instruction, assessment, and collaboration with medical educators.

## INTRODUCTION

Medical librarians have long observed a troubling pattern, that medical students who struggle with complex textbooks also demonstrate weak information literacy skills [1, 2]. Those who avoid dense scientific texts or who highlight passively, show poor source evaluation and also search unsystematically [2, 3]. Traditional library instruction has treated these as separate problems requiring separate solutions, with limited impact. What if we've been addressing symptoms rather than causes?

Before presenting this reconceptualization, it is worth clarifying two terms that anchor the discussion. By textbook literacy, we mean the discipline-specific reading competence required to engage productively with medical and scientific texts, a capacity that goes beyond general reading ability to include navigating technical vocabulary, interpreting complex textual structures, and integrating information across dense sources [1, 4]. Information literacy (IL), as defined by the Association of College & Research Libraries (ACRL) Information Literacy Framework, encompasses the reflective discovery of information, the understanding of how information is produced and valued, and the use of information in the creation of new knowledge [5].

Yet in medical education, this expansive conception of information literacy has been operationalized narrowly.

The closest IL has come to meaningful curricular recognition in medical education is through Evidence-Based Medicine, where it functions as a core clinical competency [6, 7]. But even there, instruction tends to collapse around its most visible and measurable component, database searching. This is not without reason. Searching is concrete, teachable, and maps readily onto clinical workflows. The ACRL Framework envisions something considerably broader. Its frames describe learners who treat research as iterative inquiry rather than a linear retrieval task (*Research as Inquiry*), who recognize that knowledge is produced through ongoing scholarly debate rather than deposited in authoritative sources (*Scholarship as Conversation*), and who develop critical judgment about how authority is constructed and why it varies across contexts (*Authority is Constructed and Contextual*) [5]. These are not peripheral extensions of IL; they are its conceptual core. When medical education reduces IL to search mechanics, it teaches students to retrieve information without developing the cognitive capacities needed to interrogate, situate, or synthesize it.

The shared cognitive architecture this commentary proposes is an architecture for that fuller conception of literacy. Specifically, we argue that textbook literacy and information literacy are not sequential competencies — where reading ability enables effective information engagement— but rather parallel manifestations of the same underlying cognitive foundations. Understanding this shared architecture has profound implications for how health sciences librarians design interventions, structure instruction, and position themselves as essential contributors to medical education

## THE TWO CONVERGING LITERACY CRISES

### The Challenge of Discipline-Specific Reading in Medical Education

Medical students arrive with general literacy skills adequate for undergraduate success yet consistently struggle with specialized reading demands of medical and scientific texts. This reflects the transition from general academic reading to discipline-specific literacy, a transition medical education has largely failed to explicitly address.

Medical schools operate under what researchers term a ‘problematic assumption’, that students possess the background knowledge and skills to comprehend scientific texts without targeted instruction [1, 8]. Ivanitskaya et al. argued that “medical schools have wrongly assumed that students have the background knowledge and skill to understand scientific texts at an appropriate level” [1]. Students report difficulty with technical terminology and textual structures characteristic of scientific papers; challenges stemming not from poor general reading skills but from unfamiliarity with discipline-specific conventions [1, 4]. Another factor is what researchers identify as a “paucity of reading comprehension strategies and metacognition” [9]. Students haven't been taught strategic approaches to complex scientific material.

Research on students' actual reading practices reveals the scope of the instructional gap. Students demonstrate avoidance patterns, preferring lecture notes and review materials over textbooks and primary literature. Research on the rationale for this avoidance points to several intersecting factors. In a multi-institutional study of clerkship reading habits, Kind et al. found that students identified time as the primary barrier to reading, with question banks and board review books rated as most useful for exam preparation [10]. Eighty percent of students ranked classmates and senior students as most influential in resource selection, suggesting that avoidance cultures propagate through peer networks. Dense scientific texts require readers to build, test, and revise mental models of complex physiological and pathological processes. These acts of schema construction build the organized knowledge structures that underlie expert clinical reasoning [8, 11].

This leads us to believe that when students substitute condensed review materials for sustained engagement with primary sources, they may acquire testable facts without developing the interconnected knowledge networks that support transfer to novel clinical problems. They spend minimal time with the complex texts that should form the foundation of medical knowledge, thus undermining the development of the deep, integrated understanding necessary for clinical reasoning and lifelong learning [8, 12].

### The Parallel Challenge of Information Literacy

While students struggle with textbook reading, medical educators have also observed persistent information literacy deficits despite years of library instruction. Medical students and residents demonstrate persistent information literacy deficits that extend well beyond the mechanics of database searching such as difficulty evaluating the credibility and context of sources, the inability to recognize knowledge as contested and evolving rather than fixed and retrievable, and an inability to synthesize meaningfully across multiple conflicting sources [13–16]. These deficits persist even among students with strong MCAT scores and academic records [17].

This persistence suggests that skills-based training overlooks the true nature of information literacy. From a cognitive perspective, IL is a complex form of information problem solving—a metacognitively-driven process of identifying a knowledge gap, formulating a search, and integrating new information to construct meaning [2]. Foundational LIS models, such as Kuhlthau's Information Search Process, describe this as an intensive cognitive and affective journey where learners must manage uncertainty to build new understanding [18]. Modern standards reflect this model. The *Framework for Information Literacy* posits that learners must cross conceptual thresholds, such as understanding “Searching as Strategic Exploration” (a process of self-guided inquiry) and “Authority is Constructed and Contextual” (a judgment about the nature of knowledge) [5].

In the medical context, the cognitive process is not abstract, it is the foundational engine of EBM. The seminal definition of EBM requires that physicians engage in the “conscientious, explicit, and judicious use of current best evidence in making decisions about the care of individual patients” [19]. This entire process, formulating a clinical question, searching for evidence, and critically appraising evidence, *is* an act of information literacy, one that is formally recognized as a core competency of practicing medicine [6, 7].

Therefore, a deficit in IL is not a peripheral “library skills problem,” it is a direct barrier to practicing EBM, the standard of modern medical care. It is these foundational cognitive skills, the “self-guided inquiry” and “judgment about knowledge,” that medical education must begin to teach and health sciences librarians need to better incorporate into instruction.

### The Puzzle of Co-Occurring Deficits

The puzzling question is, “Why do textbook literacy difficulties and information literacy deficits so consistently co-occur?” Both textbook literacy and information literacy are multidimensional constructs shaped by prior knowledge, disciplinary socialization, effective responses to uncertainty, and instructional context. We do not intend to reduce either to a single skill. Rather, we argue that specific cognitive capacities emerge as shared foundations that help explain patterns of co-occurring difficulty across both domains.

Traditional explanations treat these as independent problems; students who can't read well naturally can't engage effectively with information either. But this sequential explanation fails to account for key observations. Students with adequate general literacy still struggle with understanding medical texts and critically appraising the evidence they find. Interventions targeting reading alone don't improve evidence synthesis. Conversely, instruction in formulating clinical questions doesn't improve textbook comprehension.

These are not separate deficits with incidental co-occurrence, but parallel expressions of shared underlying cognitive mechanisms. This “puzzle” is largely an artifact of siloed professional disciplines. Cognitive scientists and literacy researchers have argued that the distinction between textbook literacy and information seeking is artificial in complex learning environments [20, 21]. The cognitive task of building a mental model from multiple, complex, often conflicting sources, known as multiple document comprehension, is the shared architecture, whether those documents are textbook chapters or database search results [20, 21].

### The Shared Cognitive Architecture

A reasonable objection is that the capacities described below are foundational to most forms of learning, not just reading and information practice. We concede the point. These are general cognitive capacities. What current instruction overlooks, however, is their simultaneous expression across two domains that medical education treats as separate. When a student reads a pathophysiology chapter without monitoring comprehension, that same student might search PubMed without monitoring search effectiveness. When a student is overwhelmed by the density of a pharmacology text, that same student may be overwhelmed by the task of synthesizing multiple search results. The contribution of this framework is not in identifying novel cognitive capacities but in making visible the shared architecture that connects these parallel deficits, and in providing a unified instructional rationale rather than separate remediation strategies for separate problems

Both textbook literacy and information literacy depend on three interconnected cognitive capacities. Understanding these as foundational, rather than peripheral, reframes how we approach “library” instruction in medical education. As illustrated in Table 1, the cognitive deficits students demonstrate when struggling with textbooks are functionally identical to those they demonstrate when struggling to critically appraise clinical evidence.

**Table 1:** The Shared Cognitive Architecture of Literacy Deficits: Mapping the Parallel Cognitive Demands of Reading and Searching

Cognitive Pillar	The “Textbook Literacy” Deficit	The “Information Literacy” Deficit	The Shared Instructional Solution
1. Metacognitive Self-Regulation *(Monitoring & Directing)*	The Unaware Reader: Continues reading despite loss of comprehension; fails to re-read or look up unknown terms; lacks “fix-up” strategies for confusion [47, 48].	The Unreflective Inquirer: Clicks links without planning; accepts initial results without evaluation; reads a clinical guideline without questioning its applicability; fails to recognize when an information strategy is not meeting a need [2, 38, 49].	Teach Monitoring: Require students to articulate goals before starting, periodically check if results/text match goals, and evaluate strategy effectiveness.
2. Working Memory Management *(Processing Capacity)*	The Overwhelmed Reader: Attempts to memorize isolated facts without schemas; overwhelmed by complex syntax; fails to use structural cues (headings) to chunk data [50–52].	The Overwhelmed Synthesizer Juggles sources without organizational criteria; Trial-and-error searching; opens tabs randomly; cannot hold competing claims in mind long enough to synthesize them; struggles equally when searching or appraising evidence [38, 39].	Teach Structure: Provide external scaffolds (graphic organizers, search logs, evidence appraisal templates) to offload working memory and reduce extraneous load.
3. Epistemic Sophistication *(Nature of Knowledge)*	The Fact-Seeker: Views texts as repositories of absolute truth; frustrated by nuance or conflicting physiological theories; seeks “the right answer” [46].	The Certain Knower: Seeks single authoritative sources; accepts clinical guidelines uncritically; paralyzed or dismissive when experts disagree; lacks framework for understanding why authority varies across contexts [53, 54].	Teach Complexity: Explicitly discuss evidence hierarchies, the probabilistic nature of medical knowledge, and the value of expert disagreement.

#### 1. Metacognitive Self-Regulation: Monitoring and Directing One's Own Learning

Metacognition encompasses both metacognitive knowledge (understanding what strategies work and why) and metacognitive regulation (actively monitoring and controlling learning processes) [22, 23]. Zimmerman's self-regulated learning theory demonstrates that successful learners plan, monitor, and evaluate their learning through cyclical processes of forethought, performance, and self-reflection [24]. In medical education, metacognitive skills predict academic performance better than many traditional aptitude measures [25–27], with metacognitively aware learners consistently outperforming peers even when matched on prior knowledge and general cognitive ability [28–30].

*Implication for librarians:* Teaching students to first articulate their goals, monitor whether the process is meeting their information needs, and evaluate whether to modify their approach teaches universal metacognitive practices applicable across the full range of information literacy behaviors. This is the cognitive core of *Research as Inquiry*, the recognition that engaging with information is an iterative, self-directed process of building understanding rather than a single retrievable act.

#### 2. Working Memory Management and Cognitive Load

Working memory, the cognitive system for temporarily holding and manipulating information, is a multi-component system with limited capacity [31, 32]. Building on this model, Sweller's Cognitive Load Theory [33, 34] defines three load types that can overwhelm the system: intrinsic (the task's inherent difficulty), germane (load for building schemas), and extraneous (load from poor instructional design).

The theory is rooted in our understanding that limited working memory capacity can be easily overwhelmed when processing complex information simultaneously. Recent applications to medical education demonstrate that instructional design ignoring cognitive load principles consistently produces poor learning outcomes [35–37]. When working memory is overloaded, learning cannot occur effectively regardless of motivation or prior knowledge.

These types of loads manifest in recognizable ways across both domains. Intrinsic load in textbook reading stems from the material's inherent complexity. A paragraph on cardiac electrophysiology requires holding multiple ion channel states in mind simultaneously. In information gathering, intrinsic load appears differently but analogously. Formulating a clinical question into searchable components while maintaining awareness of the clinical context requires juggling multiple conceptual elements at once. Germane load, the productive effort of building schemas, looks like a student connecting a textbook passage on renal physiology to a previously learned concept in cardiac output. In searching, it is akin to a student recognizing that two search results describe the same mechanism from different clinical perspectives and constructing a coherent model from both. Extraneous load, or wasted effort from poor design, includes unnecessarily complex textbook layouts in one domain and cluttered database interfaces in the other.

*Implication for librarians:* Teaching students to document their reasoning process, use structured evaluation criteria, and chunk complex information into manageable units teaches cognitive load management principles applicable across the full range of information literacy behaviors. This includes not only searching, but also reading primary literature, appraising conflicting evidence, and situating a source within an ongoing scholarly conversation. A student overwhelmed by the density of a pharmacology text may well be the same student who struggles to hold competing claims from multiple systematic reviews in mind long enough to synthesize them. Research suggests the cognitive bottleneck is shared even when the task looks different [32, 33, 38]. Recent studies support this approach, showing that instruction addressing cognitive load produces better outcomes than traditional skills-based training [39, 40].

#### 3. Epistemic Sophistication: Understanding Knowledge as Constructed and Contextual

Epistemic cognition refers to individuals' conceptions about the nature of knowledge and knowing. William Perry first described students' intellectual development from simplistic, dualistic views of knowledge (“there are right answers that authorities know”) to complex, contextual, committed relativism (“knowledge is constructed, contextual, and requires evidence-based judgment”) [41, 42]. This pillar is directly “braided” with metacognition. As Hofer and Pintrich argued, a student's epistemic beliefs directly influence their use of metacognitive strategies [43]. Research shows that students with naive epistemic beliefs adopt rigid, surface-level strategies in both reading and information seeking, failing to tolerate the ambiguity inherent in clinical evidence [44–46].

It is worth noting that the educational structures preceding medical school may actively reinforce this epistemic stance. The MCAT and the multiple-choice formats that dominate preclinical and licensing examinations reward single-best-answer selection, precisely the dualistic orientation that Perry identified as the earliest stage of intellectual development [41]. Students arrive having been rewarded for finding “the right answer” and are then expected to navigate a landscape of probabilistic, contextual, and often conflicting clinical evidence. Interventions aimed at developing epistemic sophistication must reckon with this structural reinforcement.

*Implication for librarians:* When we teach source evaluation, we implicitly teach epistemic concepts about evidence and knowledge construction in medicine; helping students understand why we privilege peer-reviewed literature, how evidence hierarchies work, and why expert disagreement exists. Making these concepts explicit develops epistemic sophistication applicable to both reading and searching. This is precisely what *Authority is Constructed and Contextual* and *Scholarship as Conversation* demand of learners. It isn’t merely the ability to locate a credible source, but the capacity to understand why credibility is constructed differently across contexts, and to recognize that the conflicting expert voices they encounter in the literature are not a problem to be resolved but a feature of how medical knowledge evolves. Research has argued that medical students should learn basic terminology and methods of epistemology as they're introduced to EBM concepts [13], positioning librarians as natural partners in this epistemic development.

### Synthesis: The Integrated Framework

These parallels are not coincidental; they are two manifestations of the same cognitive-constructivist process. Kintsch's *construction-integration model* posits that readers build mental representations by integrating new text with existing knowledge [11]. This mirrors the information gathering process, where students must construct coherent mental models from multiple, often conflicting, sources.

Both literacies are acts of schema construction governed by the same working memory limitations and guided by the same metacognitive judgments. Consequently, traditional interventions fail because they treat symptoms rather than causes. Teaching students to locate sources without developing the cognitive capacity to interrogate, situate, or synthesize them is like teaching vocabulary without comprehension strategies: it provides tools but not the architecture to use them.

### Practical Implications for Health Sciences Librarianship

Understanding the shared cognitive architecture of reading and information literacy has immediate practical implications for how health sciences librarians design instruction, assess student competencies, and position themselves within medical education.

### Rethinking Instructional Design

Many skilled health sciences librarians already incorporate elements of what we describe. Experienced instruction librarians routinely teach students to decompose research questions, evaluate sources critically, and reflect on search effectiveness. These practices naturally align with the cognitive architecture outlined above. However, the available evidence suggests this kind of instruction remains the exception in medical education. Smith and Sanger described their multi-year, scaffolded IL program at McMaster University as a “rarity” in undergraduate health sciences, noting that one-shot, skill-based instruction remains the dominant model despite its acknowledged limitations [55]. Similarly, Hartman et al. documented persistent reliance on inappropriate resources among medical students even after standard library instruction [3].

The framework we propose does not introduce entirely new teaching practices. It provides a theoretical language, grounded in cognitive science, that makes explicit the rationale for the kind of integrated, metacognitive instruction that effective librarians already practice. This language serves two purposes: 1) it offers a design framework for librarians moving beyond skills-based instruction, and 2) it provides the vocabulary necessary to advocate for embedded IL instruction in the terms that medical educators and curriculum committees use.

Since reading and information engagement share cognitive foundations, instruction addressing both simultaneously might be more effective than separate approaches. Library sessions could explicitly connect database searching strategies to textbook reading strategies, helping students recognize that the metacognitive monitoring skills used while reading (Do I understand this? Do I need more information? How does this connect to what I know?) apply directly to formulating a clinical question or synthesizing conflicting sources (Is my search strategy working? Do I need to modify my approach? How does this source relate to others I’ve found?).

Expert librarians execute sophisticated cognitive processes automatically, monitoring search effectiveness, managing cognitive load by chunking results into categories, and applying epistemic frameworks to evaluate sources. Novice students don't see these processes. Instructional design should make invisible cognitive processes visible through think-aloud demonstrations, metacognitive prompting, and explicit discussion of epistemic frameworks underlying source evaluation.

### Reimagining Assessment

Many information literacy assessments already incorporate measures of critical engagement with sources, and this represents important progress beyond simple task completion. The cognitive architecture framework suggests a further extension, assessing not only whether students engage critically, but also questioning the underlying processes that enable that engagement. A student may identify a high-quality systematic review while relying on a surface-level heuristic (choosing the first Cochrane result) rather than genuine epistemic reasoning about evidence hierarchies. Process-level assessment asks not only “Did the student find an appropriate source?” but “Can the student articulate why it is appropriate, monitor whether their search strategy is working, and recognize when to change approach?”

If reading comprehension and database searching share cognitive foundations, information literacy assessments should also include strategic reading assessments. Students who cannot effectively read and integrate information from scientific texts fundamentally lack a core information literacy competency, even if they can execute database searches. This provides an opportunity to reframe how the scope of information literacy in medical education is conceptualized.

### Positioning Librarians in Medical Education

Understanding that information literacy depends on fundamental cognitive capacities positions librarians not only as service providers teaching technical skills, but also as educational partners addressing core learning competencies. When a medical educator observes that students “can't evaluate sources,” it may reflect a deficit in metacognitive monitoring, cognitive load management, and epistemic sophistication, the same deficits resulting in reading comprehension difficulties. This positions library instruction to address fundamental educational objectives rather than only peripheral research skills.

Many medical educators recognize that students struggle with reading comprehension but feel ill-equipped to address it. Librarians, equipped with this cognitive framework, can partner with medical educators to develop integrated interventions addressing both reading and information practice. Problem-based learning sessions could incorporate explicit instruction in metacognitive monitoring applicable to both textbook study and evidence searching. Clinical reasoning exercises could explicitly address epistemic sophistication in ways supporting both clinical decision-making and information evaluation.

When advocating for information literacy instruction, librarians can articulate their value in the cognitive and educational language that medical educators use. Rather than arguing that students need to “learn how to utilize PubMed," students instead need to develop metacognitive self-regulation, manage cognitive load in complex information environments, and develop epistemic sophistication about medical evidence. These cognitive capacities serve as foundational competencies that can help predict future clinical competence and lifelong learning capability.

### Future Directions and Research Questions

This reconceptualization opens several research questions that health sciences librarians are primed to pursue. Intervention studies comparing integrated cognitive instruction against traditional skills-based sessions would test whether the framework improves outcomes across both reading and information engagement. Librarians designing new IL programs could embed such comparisons within existing instruction. Transfer studies would test whether metacognitive strategies learned in one domain (e.g., search strategy monitoring) would improve performance in the other (e.g., textbook comprehension), validating the shared architecture claim and informing instructional design. Finally, practical assessment instruments measuring metacognitive monitoring, cognitive load management, and epistemic reasoning could identify at-risk students early and target interventions before deficits compound.

## CONCLUSION

Understanding this shared cognitive architecture has immediate practical implications for instructional design, assessment, and positioning within medical education. Rather than teaching discrete information skills in isolation and hoping students will transfer them, students should be taught the underlying cognitive strategies enabling both effective reading, searching, and critical engagement of evidence. Rather than positioning librarians as service providers teaching peripheral technical skills, they should be considered educational partners addressing fundamental learning competencies that predict clinical success.

In most medical schools, information literacy instruction currently takes the form of limited, session-based training focused on database mechanics, search syntax, and source identification [3, 55]. Even when these sessions incorporate critical evaluation, they are rarely integrated into the broader medical curriculum, rarely scaffolded across preclinical and clinical years, and rarely designed with explicit attention to the cognitive processes that determine whether students can transfer what they learn. The framework proposed here envisions a concrete shift. From isolated sessions to longitudinal cognitive instruction embedded within medical courses, from teaching search mechanics to teaching metacognitive monitoring as a transferable clinical skill, and from assessing whether students can complete a search to assessing whether they can regulate their own learning across both domains. This shift does not require librarians to abandon existing expertise but to reframe it in terms that articulate its value to medical educators and curriculum committees.

The evidence supporting this framework comes from multiple disciplines including cognitive psychology's models of reading comprehension and information processing, educational research on metacognition and self-regulated learning, medical education research on clinical reasoning development, and information science research on information-seeking behavior. By integrating insights from these fields, educators can move beyond treating symptoms and address the underlying causes.

This reconceptualization doesn't diminish the importance of technical skills like Boolean operators or reading techniques like SQ3R. Rather, it situates these technical skills within a broader cognitive framework that explains when, how, and why to deploy them. Students need both the cognitive architecture and the technical tools, but without the architecture, the tools remain inert.

Medical education faces increasing complexity, expanding knowledge bases, proliferating information sources, growing emphasis on evidence-based practice, and emerging challenges from AI-generated content. In this environment, the fundamental cognitive capacities described become more critical, not less. Students must learn not just how to think, but how to monitor their understanding, manage cognitive complexity, and evaluate knowledge claims in an uncertain, evolving evidence landscape.

Health sciences librarians are ideally positioned to lead in developing these capacities. They work at the intersection of information, learning, and evidence-based practice. These librarians understand both the technical requirements of information access and the cognitive requirements of information synthesis. By embracing this broader understanding of a librarian’s educational role, not as purveyors of database skills but as developers of fundamental cognitive competencies, instruction can be made more effective, assessment more meaningful, and value more apparent to medical educators and students alike.
